# Correspondence for the article titled: “Impact of uncomplicated traumatic dental injuries on the quality of life of children and adolescents: a systematic review and meta-analysis”

**DOI:** 10.1186/s12903-021-01493-4

**Published:** 2021-03-19

**Authors:** Nitesh Tewari, Gangadharao Morankar Rahul, Mugilan Ravi

**Affiliations:** Division of Pedodontics and Preventive Dentistry, 6th Floor, Centre for Dental Education and Research, All India Institute of Medical Sciences, New Delhi, 110029 India

**Keywords:** Oral health related quality of life, Children, Traumatic dental injuries, Systematic review

## Abstract

As part of the critical appraisal exercise in our center, we reviewed the article published in BMC Oral Health, titled "Impact of uncomplicated traumatic dental injuries on the quality of life of children and adolescents: a systematic review and meta-analysis" authored by Lopez et al. (BMC Oral Health 19(1):224, 2019). We noted a plausible error that can influence the outcomes of this systematic review and meta-analysis which necessitates interpreting the findings of this systematic review with caution.

## Background

An article titled "Impact of uncomplicated traumatic dental injuries on the quality of life of children and adolescents: a systematic review and meta-analysis" authored by Lopez et al. [1] was published in your esteemed journal BMC Oral Health. It had concluded that uncomplicated Traumatic Dental Injuries (TDI) do not have a negative impact on the Oral Health-Related Quality of Life (OHRQoL) of children and adolescents. It was an interesting and enriching systematic review to read and we would congratulate the authors for following the best practices for evidence-based medicine. This is an unexplored area of dental traumatology and requires better understanding.

## Discussion

During the critical appraisal of the article, we identified a discrepancy that is capable of influencing the outcomes of this systematic review and meta-analysis. Two of the included primary studies by Neves et al. [2] and Gomes et al. [3] had been conducted by the same research group in the same year (2017). It was further noted that both the studies had several other identifying characteristics such as the ethics committee's approval number, the study population, the sample size, and the tool used for assessment of outcome (SOHO-5) [4]. Though, both the articles were published in different journals, they represent the same data. We don't know the exact reason behind the duplication, however, the identical sample population, time period, and other characteristics are difficult to ignore.

Since the authors of the systematic review could not identify this duplication and included the data from both the studies, they ended up giving more weight to the outcomes from the same population. This error can further increase the bias in the meta-analysis and hence the conclusions of the review which can be rendered misleading. In the course of performing a systematic review, it is always a good practice to seek clarifications from the authors of primary studies which exhibit ambiguity. Another astounding observation was that these primary studies [1, 2] had been weighted differently for different outcomes in the forest plots and showed the different risk of bias (New Castle Ottawa Scale) even after having identical characteristics. The findings of this Systematic Review should be interpreted with caution as the inclusion of both these studies can modify the estimated effect which could be a cause of concern for the researchers working in this field.

## Data Availability

Not applicable.

## Abbreviations

TDI: Traumatic dental injuries
OHRQoL: Oral health related quality of life
SOHO: Scale of oral health outcomes

## References

[1] Lopez D, Waidyatillake N, Zaror C, Mariño R (2019). Impact of uncomplicated traumatic dental injuries on the quality of life of children and adolescents: a systematic review and meta-analysis. BMC Oral Health.

[2] Neves ETB, Perazzo MF, Gomes MC, Martins CC, Paiva SM, Granville-Garcia AF (2017). Perception of parents and self-reports of children regarding the impact of traumatic dental injury on quality of life. Dent Traumatol.

[3] Gomes MC, Perazzo MF, Neves ÉT, Martins CC, Paiva SM, Granville-Garcia AF (2017). Oral problems and self-confidence in preschool children. Braz Dent J.

[4] Tsakos G, Blair YI, Yusuf H, Wright W, Watt RG, Macpherson LM (2012). Developing a new self-reported scale of oral health outcomes for 5-year-old children (SOHO-5). Health Qual Life Outcomes.

